# Polypropylene Degradation on Co-Rotating Twin-Screw Extruders

**DOI:** 10.3390/polym15092181

**Published:** 2023-05-04

**Authors:** Matthias Altepeter, Volker Schöppner, Sven Wanke, Laura Austermeier, Philipp Meinheit, Leon Schmidt

**Affiliations:** Kunststofftechnik Paderborn, Paderborn University, 33098 Paderborn, Germany

**Keywords:** twin-screw-extrusion, polypropylene, molar degradation, compounding

## Abstract

Nowadays, usable plastic materials with defined properties are created by blending additives into the base polymer. This is the main task of compounding on co-rotating twin-screw extruders. The thermal and mechanical stress occurring in the process leads to a mostly irreversible damage to the material. Consequently, the properties of the polymer melt and the subsequent product are affected. The material degradation of polypropylene (PP) on a 28 mm twin-screw extruder has already been studied and modeled at Kunststofftechnik Paderborn. In this work, the transferability of the previous results to other machine sizes and polypropylene compounds were investigated experimentally. Therefore, pure polypropylene was processed with screw diameters of 25 mm and 45 mm. Furthermore, polypropylene compounds with titanium dioxide as well as carbon fibers were considered on a 28 mm extruder. In the course of the evaluation of the pure polypropylene, the melt flow rates of the samples were measured and the molar masses were calculated on this basis. The compounds were analyzed by gel permeation chromatography. As in the previous investigations, high rotational speeds, low throughputs and high melt temperatures lead to a higher material degradation. In addition, it is illustrated that the previously developed model for the calculation of material degradation is generally able to predict the degradation even for different machine sizes by adjusting the process coefficients. In summary, this article shows that compounders can use the recommendations for action and the calculation model for the material degradation of polypropylene, irrespective of the machine size, to design processes that are gentle on the material.

## 1. Introduction

To produce usable plastics, the properties of the materials must be specifically modified. This is conducted during compounding on co-rotating twin-screw extruders. Additives such as fillers, fibers or colors are mixed with the base polymer. Besides the desired melting and homogenization of the components, the thermal and mechanical stress in the extruder causes mostly irreversible damage to the material. The occurring damage mechanisms can be divided into purely thermal, thermal–oxidative, thermal–mechanical and hydrolytic degradation. These reactions usually run simultaneously and lead to different types of damage. Mainly, chain scission results in a reduction in the molar mass and a change in the molar mass distribution. This influences the rheological properties of the melt in the process as well as the mechanical properties such as the tensile strength of the end product. Moreover, color or odor changes may occur [1,2,3].

The evaluation of the material degradation caused by the compounding process, which is usually only possible retrospectively, is associated with a high economic and time expenditure, since a suitable operating point must first be detected using a try and error procedure. This problem led to the objective of a research project carried out at Kunststofftechnik Paderborn (KTP). It pursues the development of recommendations for gentle processing as well as mathematical modeling to describe the material degradation of market-dominating polypropylene on the co-rotating twin-screw extruder.

The literature already contains several studies on the material degradation of PP on single- or twin-screw extruders. Pongratz [4], for example, investigated the thermal, thermal–oxidative and mechanical degradation of polyamide (PA) and PP during processing and in use. It was demonstrated that the material degradation of PP during the twin-screw extrusion is significantly influenced by the process parameters and the atmosphere. Low processing temperatures, low rotational speeds, high throughputs and inert atmospheres result in a low molecular damage and a high oxidation induction time. Accordingly, there is little material degradation. However, the significance of the results is limited because only low rotational speeds (50 and 300 rpm) and low throughputs (3 and 6 kg/h) were observed. In addition, only one twin-screw extruder with a screw diameter of 34 mm was used. The occurring material degradation of PP in the presence of peroxide on the twin screw extruder was investigated and modeled by Berzin et al. [5]. It was shown that a low throughput leads to a high residence time and thus to faster and stronger degradation. An increase in rotational speed lowers the residence time and raises the mass temperature. Since the influence of the higher melt temperature is predominant, the material degradation is increased. Due to the strong influence of the continuously used peroxide on the chain degradation, it is difficult to transfer the results to the extrusion of pure PP or other PP compounds. Canevarolo [6] analyzed the molar mass distributions of PP and found out that the probability of chain scission during processing on the twin-screw extruder depends on the number of extrusion cycles, the screw configuration and the molar mass of the chain. Chain scissions become more frequent as the number of extrusions increases. In addition, 90° kneading elements cause higher material degradation than conveying elements. Further investigations by Canevarolo and Babetto [7] confirmed the influence of the number of extrusions and the screw aggressiveness on material degradation. Da Costa et al. [8] considered the material degradation of PP during multiple extrusions on a single screw extruder based on the changes in rheological properties. They observed that high temperatures and multiple extrusions lead to a decrease in viscosity and loss of elasticity of the melt. The residence time, melt temperature and shear rate are reported as important factors in determining the degradation rate. In additional investigations, Da Costa et al. [9] focused on the thermal and mechanical properties of the extrusion products. A differential scanning calorimetry (DSC) analysis pointed out that the degree of crystallization rises with the increasing melt temperature and growing number of extrusions, whereas the melting temperature decreases. The yield strength and elastic modulus are only slightly affected by processing, whereas break properties (break stress, break strain and energy to break) are strongly reduced. The described publications provide important approaches for the investigation of the material degradation of PP on the twin-screw extruder. However, they neither offer a comprehensive investigation of all influencing factors nor a consideration of the interaction of the individual factors.

In previous research projects at the KTP, the material degradation of polyolefins on the single-screw extruder [10,11,12] and the material degradation of polyethylene (PET) on the twin-screw extruder were investigated [13,14]. Thereby, a mathematical model for predicting the molecular weight change of PP and PS during processing on the single-screw extruder has already been developed. However, due to various factors such as a deviating channel geometry and deviating shear rates, a direct transfer of the model to the twin-screw extruder is not reasonable.

Within the research project on the material degradation of PP on the twin-screw extruder, basic investigations were first carried out [15,16]. Two different PP types were processed on a ZE 28 BluePower extruder from KraussMaffei with a screw diameter of 28 mm. The results demonstrated that high rotational speeds, low throughputs and high melt temperatures lead to higher material degradation. Compared to these three influencing parameters, it was found that the different screw configurations investigated had a comparatively lower influence on the extent of degradation. This is due to the fact that different screw elements have less influence on the residence time, temperature and shear rates than the change of one of the previously mentioned process parameters (screw speed, temperature and throughput). Based on the collected data, a model was developed to describe the material degradation, which is based on the following equation:

(1) $$\frac{{\bar{M}}_{w}}{{\bar{M}}_{w,0}}=1/{\left[\exp\left(\frac{T}{23,823.97\;{}^{{}^{\circ}}C}\right)\cdot \left(1+{\left(\frac{{\dot{\gamma }}_{\mathrm{res}}}{{1219.07\;s}^{-1}}\right)}^{2}\right)\right]}^{\frac{∆t_{v}}{11.29\;s}}$$

The left side of the equation contains the weight-average molar mass to be calculated, ${\bar{M}}_{w}$, and the weight-average initial molar mass, ${\bar{M}}_{w,0}$. On the right side, there are the influencing parameters’ temperature, T, weighted shear rate, ${\dot{\gamma }}_{\mathrm{res}}$, and residence time, $∆t_{v}$, as well as the associated process coefficients. To simulate a compounding process, the SIGMA software divides the entire process into 150 calculation sections, whereby a large number of different process parameters such as pressure, filling degree or degree of melting are calculated in advance for each of these so-called supporting points. The three parameters needed to calculate the material degradation temperature, surface-weighted shear rate and residence time difference between two calculation points are also already determined during the simulations. The calculation of the material degradation takes place after the iterative simulation of the process itself by passing the required process parameters to the equation for the calculation of the material degradation at each of the support points. In this way, not only the final material degradation at the exit of the extruder but also more than the entire process can be visualized in SIGMA. The process coefficients were also determined on the basis of the experimental investigations using an Excel solver in such a way that there is a minimal deviation between the experimental and calculated material degradation coefficients. Since the model predicts the material degradation of PP on the 28 mm extruder with good accuracy, it was implemented in the simulation software of co-rotating twin screw extruders named SIGMA and developed at KTP.

This article explains the further investigations to verify the previously developed recommendations for gentle processing and the applicability of the model to varying machine sizes and PP compounds. For this purpose, the following section first explains the tests carried out on pure polypropylene on a 25 mm and 45 mm extruder, as well as studies on compounds with titanium dioxide (TiO_2_) and carbon fibers (CF). The measuring methods used to determine the material degradation are then presented, as well as recommendations for gentle processing. In addition, it is illustrated that the introduced calculation model is also generally transferable to varying plant sizes.

## 2. Materials and Methods

### 2.1. Used Materials

The PP investigated is the commercial pure polymer PP 500P from the polymer manufacturer SABIC. The multi-purpose material is suitable for applications in the fields of extrusion, injection molding and thermoforming [17]. Only material from one batch was used in the experiments to prevent the influence of batch fluctuations on the results. For the first compound, a mass fraction of 10% titanium dioxide was added to the PP as a dry blend before extrusion. The CR-470 from Tronox is used for high-temperature PP extrusion, rigid and flexible vinyl products and polyolefin or ABS masterbatches [18]. The second compound was made by adding a mass fraction of 20% of carbon fibers to the PP by side feeding after the melting zone. IM-OX-05/12 short fibers from C.A.R. FiberTec GmbH were used. Among other applications, these are suitable for the aerospace sector due to their high strength and low density [19]. Table 1 provides an overview of the material properties specified by the manufacturers.

### 2.2. Processing

**Figure 1:**
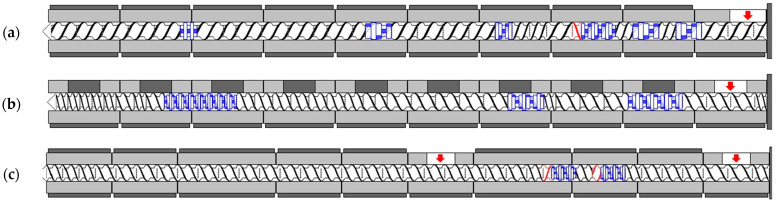
Screw configurations of the 25 mm (**a**), 45 mm (**b**) and 28 mm twin-screw extruder (**c**).

Two twin-screw extruders with screw diameters of 25 mm (Coperion ZSK 25 P8) and 45 mm (Barmag Saurer BTS-PET-045/40D) were used to investigate the transferability of the developed degradation model to other machine sizes. The PP compounds were processed on a ZE 28 BluePower twin-screw extruder from KraussMaffei with a screw diameter of 28 mm. Since the screw configuration can be assumed to have a small influence on the material degradation according to the findings of the previous studies, only one screw configuration was considered for each setup (Figure 1). The configurations mainly consist of conveying elements downstream of the plasticizing zone.

**Figure 2:**
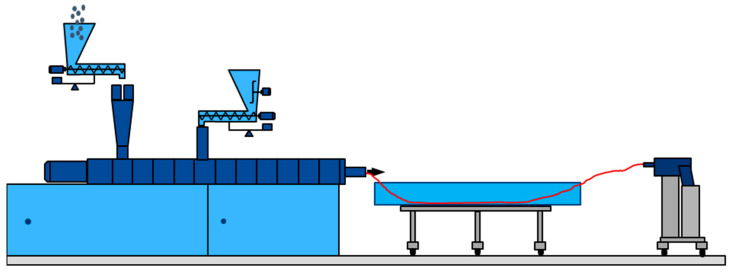
Schematic test setup with extruder, dosing unit, water bath and pelletizer.

Gravimetric feeding and a water bath were used for all experimental investigations. The cooled extrudates were granulated with the help of a strand pelletizer. Figure 2 shows a schematic illustration of the test set-up, which was identical on all systems apart from the extruder size for the sake of comparability. In the experiments with pure polypropylene as well as the compound with titanium dioxide, all materials were dosed through the main hopper. For compounding the carbon fiber, an additional side feeder was used in zone 5. The arrows in the illustrations of the screw configurations represent the respective material feed points.

A comprehensive examination of the influence of the process parameters’ rotational speed, and the throughput and barrel temperature on material degradation was ensured by full-factorial test designs. Three different rotational speeds and three different throughputs were varied for each investigation according to Table 2. When selecting the rotational speeds, the maximum recommended circumferential speed of the materials had to be considered. The throughputs were adjusted in such way that a torque utilization of the extruder of 80% was not exceeded at the test points with the highest throughput and lowest rotational speed.

To assess the effects of the temperature configuration on material degradation, low, medium and high barrel temperatures were considered in each investigation (Table 3). While the first temperature configuration is identical in all cases, temperature configurations two and three were each adjusted. This was necessary to prevent excessive decomposition of the PP and excessive damage to the CF.

### 2.3. Measurement

As already described in section one, processing on the twin-screw extruder leads to molar mass degradation and thus to a reduction in melt viscosity. Knowledge of the quickly measurable and evaluable melt flow rate as a dimension for the viscosity therefore allows conclusions about the occurred material degradation. For this reason, the melt mass flow-rate (MFR) and the melt volume flow-rate (MVR) of the taken granulate samples were measured according to [20] using a Mflow BMF-001 extrusion plastometer from ZwickRoell. Here, the mass and volume extruded in a certain time under material-specific conditions through a standardized tool are determined. A test load of 2.16 kg and a test temperature of 230 °C were used for all measurements. The cylinder of the extrusion plastometer was filled with a sample mass of 4.0 to 4.1 g in each case.

**Figure 3:**
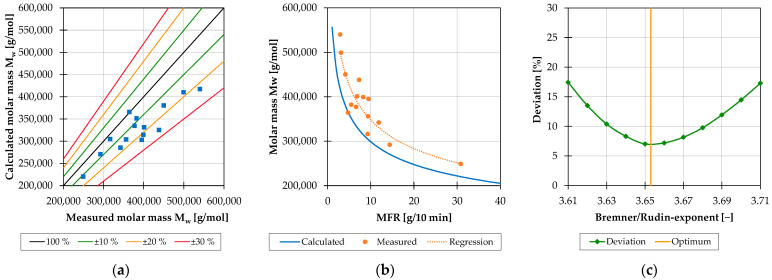
Deviation of the calculated from the measured weight-average molar masses (**a**); weight-average molar masses depending on the MFR value (**b**); mean percentage deviation of the calculated from the measured values as a function of the Bremner/Rudin exponent (**c**).

For the evaluation of the preliminary basic investigations carried out on the 28 mm extruder, the measured MFR values were converted into weight-average molar masses by using a model developed by Bremner et al. [21]. To verify these calculated values, selected test points were analyzed by gel permeation chromatography (GPC). Here, a polymer solution is pumped through porous separation columns. While large macromolecules can only settle in a few large pores, smaller macromolecules pass through a larger number of smaller pores and therefore they need more time to flow through the column. After separation, the sample is characterized using detectors. In this case, the PP samples were mixed with 1,2,4-trichlorobenzene (TCB) and dissolved at 160 °C for two hours. Furthermore, a sample concentration of 3.0 g/L, an injection volume of 200 μL, a flow rate of 1.0 mL/min as well as an infrared detector were used. The results demonstrated that the calculated weight-average molar masses consistently deviated downwards from the measured weight-average molar masses, with only one exception (Figure 3a). To shift the curve shown in Figure 3b to a higher molar mass level and thus reduce the mean percentage deviation of 14.47%, the equation was adjusted. The Bremner/Rudin exponent at which a minimum mean deviation of the calculated values from the measured values occurs was determined according to Figure 3c. This amounts to 6.93% for an exponent of 3.653. Consequently, the MFR values measured during the further investigations for the pure PP were converted into weight-average molar masses with the help of the adjusted equation:

(2) $${\bar{M}}_{w}=\sqrt[{3.653}]{1.8095\cdot 10^{21}\cdot {\mathrm{MFR}}^{-1}}$$

Since the calculation with Equation (2) is only suitable for pure polymers, the PP compounds were analyzed by GPC. This provides the advantage that the molar mass distribution is determined in addition to the weight-average molar mass. Essentially, the measurement conditions corresponded to those of the previously performed verification.

## 3. Results

### 3.1. Experimental Results

**Figure 4:**
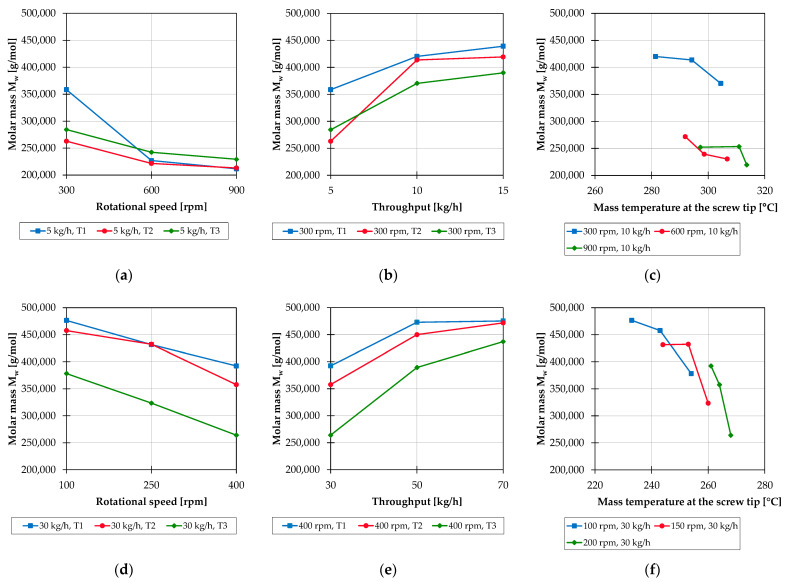
Molar mass as a function of the parameters’ rotational speed (**a**), throughput (**b**) and mass temperature (**c**) for pure PP processed on the 25 mm extruder; molar mass as a function of the parameters’ rotational speed (**d**), throughput (**e**) and mass temperature (**f**) for pure PP processed on the 45 mm extruder.

The results of the further investigations largely correspond to those of the basic investigations on the 28 mm extruder. Figure 4 shows the molar mass as a function of rotational speed, throughput and mass temperature for pure PP processed on the 25 mm extruder (a–c) and on the 45 mm extruder (d–f).

An increase in screw speed with otherwise constant parameters generally increases material degradation on all twin-screw extruder sizes. The reasons for this are the higher mechanical stress due to the increasing shear rates and the associated increased temperature of the material, which, for example, outweigh the material-protecting effect of the reducing residence time [22]. In addition, a saturation of the effect occurs at a certain operating point, from which a further increase in speed has a smaller influence on the molar mass degradation. The increasing throughput is accompanied by a lower material degradation at different machine sizes. One of the reasons for this is that the residence time in the process is shortened due to the higher throughput. Due to the increasing specific filling level, there is also less oxygen in the system; thus, the thermal-oxidative degradation is inhibited. If the melt temperature is increased by changing the temperature profile in the process, this tends to lead to increasing material damages. This is caused by the increased thermal and thermal-oxidative degradation mechanisms of the polypropylene, which lead to a reduction in the weight-average molecular weight. The mechanisms described above are explained in more detail below, where the results for the PP compounds are explained.

**Figure 5:**
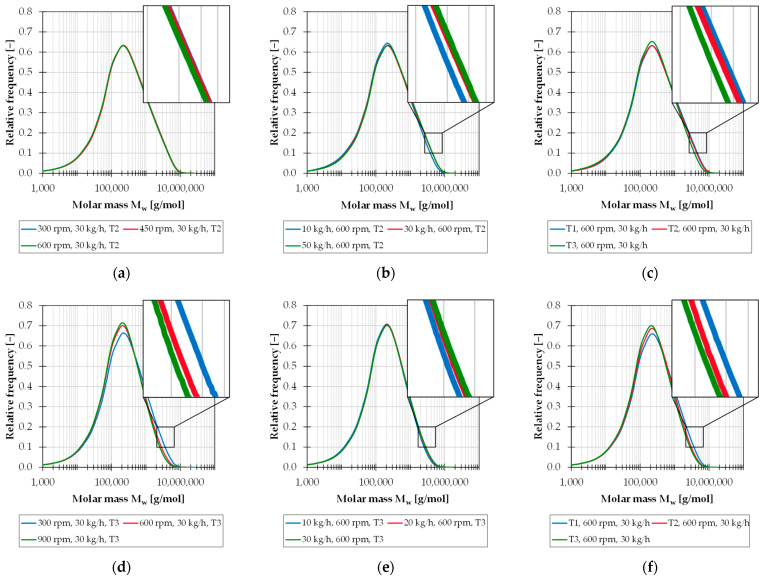
Molar mass distributions of the PP/TiO_2_ compound for different rotational speeds (**a**), throughputs (**b**) and mass temperatures (**c**); molar mass distributions of the PP/CF compound for different rotational speeds (**d**), throughputs (**e**) and mass temperatures (**f**).

The effect of the individual process parameters on the PP compounds is illustrated in Figure 5. Here, the molar mass distributions of the PP/TiO_2_ compound (a–c) and the PP/CF compound (d–f) are shown for different rotational speeds, throughputs and mass temperatures. The chain scissions caused by the processing on the twin-screw extruder leads to a shift of the distribution curves into the range of lower molar mass. Higher stress on the material due to high rotational speeds, low throughputs or high melt temperatures results in increased chain scissions and thus in a more significant displacement of the molar mass distribution.

As explained, an increase in speed causes a higher shear stress, which in turn causes a higher mechanical degradation. Here, a deformation of the bond angles and the bond distance and consequently a breakage of the molecular chain occurs. Due to the structural viscosity of PP, the higher shear stress also causes the chains to stretch and align more, so that less energy is required for the flow. This reduces the effects of mechanical damage when the rotational speed is steadily increased. The higher the shear stress and the resulting dissipation, the more the melt heats up. This heating primarily causes chain scissions through the purely thermal and especially the thermal-oxidative degradation of the material. In terms of the pure thermal degradation, PP is relatively stable. However, it reacts easily with oxygen and radicals and therefore has only a low resistance to thermal-oxidative degradation. The thermal-oxidative degradation proceeds autocatalytically, meaning the initially slow reaction is accelerated by the formation of hydroperoxides and their decomposition into radicals. In addition, the thermal-oxidative degradation is accelerated by primary radicals which are formed during mechanical degradation. The increase in rotational speed also reduces the residence time of the material in the extruder, which is gentle on the material. Since the damaging effects associated with the increase in rotational speed dominate, an overall rise in material degradation takes place [3,23].

With increased throughput comes a reduction in the residence time of the material in the extruder. This particularly reduces the mechanical degradation. The higher filling level in the channel also has a positive effect. In combination with the shorter residence time, it leads to a lower melt temperature. As a result, the purely thermal and thermal-oxidative degradation is reduced. The latter is also decreased; due to a high filling level, less oxygen is available for the oxidation of the melt. Accordingly, the higher the throughput, the lower the material degradation. Raising the barrel temperature leads to a higher mass temperature. This in turn boosts the purely thermal as well as the thermal-oxidative degradation. At the same time, a higher mass temperature is accompanied by a lower viscosity, which reduces the mechanical degradation. However, overall, there is a clear increase in material degradation. In general, the material degradation is increased by high rotational speeds, low throughputs and high melt temperatures.

### 3.2. Modeling

**Figure 6:**
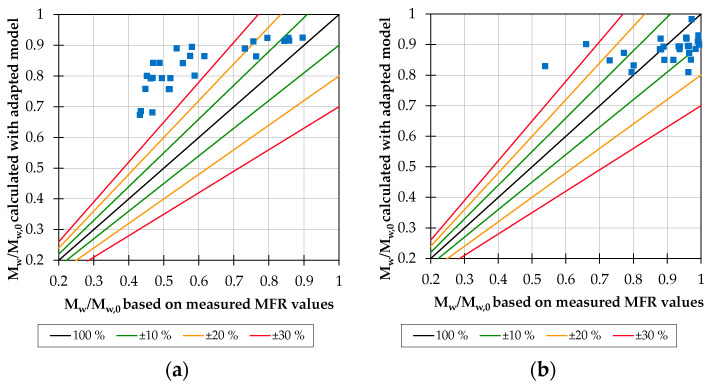

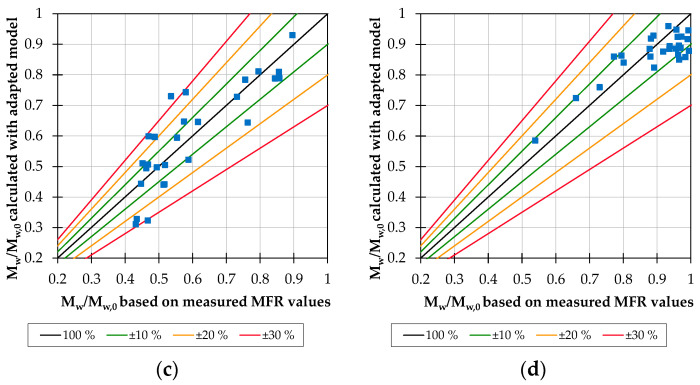
Deviation of the modeled molar mass quotients from the molar mass quotients based on the measured MFR values for pure PP on the 25 mm extruder (**a**) and the 45 mm extruder (**b**); improved accuracy of the model for the 25 mm extruder (**c**) and the model for the 45 mm extruder (**d**) through adaption of the process coefficients.

The result of the modeling with Equation (1) is the ratio of the reduced weight-average molar mass M_w_ at the particular operating point and the weight-average initial molar mass M_w,0_. If no material degradation occurred, this molar mass quotient equals one. The higher the material degradation, the more the molar mass quotient approaches zero. For pure PP on the 25 mm extruder, all modeled values show an upward deviation according to Figure 6a. The deviation is greater for high material degradation than for low material degradation. One reason for the partially higher deviations is that the melt temperatures at some operating points were above the processing or decomposition range and consequently a disproportionately large material degradation takes place. For the 45 mm extruder, lower processing temperatures were used to stay within the recommended processing temperature at all test points. Consequently, the predicted values are more accurate (Figure 6b). Considering only the test points within the processing temperature for the 25 mm extruder, the accuracy would therefore also increase.

An adjustment of the process coefficients of the model according to Table 4 leads to an improved quality of the prediction. Instead of determining the process coefficients on the basis of the investigations on the 28 mm extruder, they were determined on the basis of the experimental investigations on the 25 mm and 45 mm extruder with the help of an Excel solver to ensure that there is a minimum deviation between the experimental and calculated material degradation coefficients for the particular extruder. As illustrated in Figure 6c, the modeled molar mass ratios for the 25 mm extruder demonstrate a more balanced scatter around the 100 % line. For the 45 mm extruder, the material degradation at operating points with formerly high deviations is modeled accurately with the new process coefficients (Figure 6d). After adjusting the process coefficients for the individual machine sizes, the average total deviation of 12.6% for the 25 mm extruder and 6.5% for the 45 mm extruder is now within an acceptable range. With the 25 mm extruder, almost all points are within a 30% error band. Excluding the extreme points, for example when the recommended processing temperature is exceeded, the error band is reduced to 20%. As explained, all test points on the 45 mm extruder are within the recommended processing range, thus the error band is also significantly lower, at around 13%.

## 4. Discussion

From the previously conducted investigations on the material degradation of PP on a 28 mm twin-screw extruder, recommendations for gentle processing have already been derived. Additionally, a mathematical model for describing the material degradation was developed and implemented in the SIGMA simulation software. In this work, further experimental investigations were carried out to ensure the transferability of the previous results to other machine sizes as well as PP compounds. On the one hand, pure PP was processed on a 25 mm and a 45 mm twin-screw extruder. On the other hand, PP/TiO_2_ and PP/CF compounds were produced on a 28 mm twin-screw extruder. For the pure PP, the melt flow rates were measured and the molar masses were calculated. Since the calculation of the molar mass from the MFR is only suitable for pure polymers, the compounds were analyzed by GPC. The results correspond to the findings obtained for the 28 mm extruder and to the conclusions in the literature [4,5] on the influence of the process parameters on the material degradation of PP. In general, high speeds, low throughputs and high melt temperatures lead to greater material degradation, independent of the machine diameter and also when processing different PP compounds. The higher the stress on the material and thus the quantity of chain scissions, the more the distribution curves shift to a lower molar mass level. Since it has been demonstrated in this article that the recommendations for action for material-friendly processing are independent of the machine size or compound, the compounder is enabled to apply them generally in the event of excessive material degradation in the process. After the adjustment of the process coefficients for the different machine sizes, the developed basic model provides a reliable prediction of the material degradation of pure PP on the 25 mm and 45 mm extruder. For the future, investigations are planned on even more different plant sizes and with varying compounds in order to further verify the results. After the completion of all investigations, a final adaptation of the model should enable a calculation of the material degradation of PP on different twin-screw extruder sizes that is as generally valid as possible. This will enable compounders to quantify the material degradation in advance and to reduce time and costs as well as to improve the product quality by finding the optimal process point.

## Figures and Tables

**Table 1:** Properties of the investigated materials [17,18,19].

Material Property	PP	TiO_2_	CF
Density [g/cm^3^]	0.905–0.93	-	1.78
Bulk density [g/cm^3^]	-	0.7	0.36–0.46
Melt mass flow rate [g/10 min]	3.1	-	-
Melting temperature [°C]	160–170	-	-
Processing temperature [°C]	235–250	-	-
Decomposition temperature [°C]	300	-	-
Average particle size [μm]	-	0.2	-
Fiber length [mm]	-	-	4
Filament number [K]	-	-	12
Filament diameter [μm]	-	-	5

**Table 2:** Rotational speeds and throughputs considered in the investigations.

Material	Extruder [mm]	Rotational Speed [rpm]			Throughput [kg/h]		
PP	25	300	600	900	5	10	15
PP	45	100	250	400	30	50	70
PP/TiO_2_	28	300	450	600	10	30	50
PP/CF	28	300	600	900	10	20	30

**Table 3:** Investigated temperature configurations.

Material	Extruder [mm]	Temperature Configuration	T_Z1_ [°C]	T_Z2_ [°C]	T_Z3_ [°C]	T_Z4_ [°C]	T_Z5_ [°C]	T_Z6_ [°C]	T_Z7_ [°C]	T_Z8_ [°C]	T_Z9_ [°C]	T_Z10_ [°C]
All investigations		T1	20	180	200	220	220	220	220	220	220	220
PP	25	T2	20	205	225	245	245	245	245	245	245	245
		T3	20	230	250	270	270	270	270	270	270	270
PP	45	T2	20	195	215	235	235	235	235	235	235	235
		T3	20	210	230	250	250	250	250	250	250	250
PP/TiO_2_	28	T2	20	230	250	250	270	270	270	270	270	250
		T3	20	280	300	300	320	320	320	320	320	300
PP/CF	28	T2	20	205	225	225	245	245	245	245	245	235
		T3	20	230	250	250	270	270	270	270	270	250

**Table 4:** Adaption of the process coefficients of the degradation model for pure PP.

Process Coefficients	Before Adaption	After Adaption	
		25 mm Extruder	45 mm Extruder
T_0_ [°C]	23,823.97	23,278.54	931.81
${\dot{\gamma }}_{0}{\;[s}^{-1}]$	1219.07	741.84	16,809.61
t_v,0_ [s]	11.29	8.75	4.5

## Data Availability

Not applicable.
